# Measuring changes in alcohol use in Finland and Norway during the COVID‐19 pandemic: Comparison between data sources

**DOI:** 10.1002/mpr.1892

**Published:** 2021-08-27

**Authors:** Pia Mäkelä, Ingeborg Rossow, Inger Synnøve Moan, Elin K. Bye, Carolin Kilian, Kirsimarja Raitasalo, Peter Allebeck

**Affiliations:** ^1^ Finnish Institute for Health and Welfare Helsinki Finland; ^2^ Norwegian Institute of Public Health Oslo Norway; ^3^ Institute of Clinical Psychology and Psychotherapy Technische Universität Dresden Dresden Germany; ^4^ Department of Global Public Health Karolinska Institutet Stockholm Sweden

**Keywords:** alcohol consumption, COVID‐19 pandemic, data sources, measurement

## Abstract

**Objectives:**

To examine (1) how a rapid data collection using a convenience sample fares in estimating change in alcohol consumption when compared to more conventional data sources, and (2) how alcohol consumption changed in Finland and Norway during the first months of the COVID‐19 pandemic.

**Methods:**

Three different types of data sources were used for the 2nd quarter of 2020 and 2019: sales statistics combined with data on unrecorded consumption; the rapid European Alcohol Use and COVID‐19 (ESAC) survey (Finland: *n* = 3800, Norway: *n* = 17,092); and conventional population surveys (Finland: *n* = 2345, Norway: *n*1 = 1328, *n*2 = 2189, *n*3 = 25,708). Survey measures of change were retrospective self‐reports.

**Results:**

The statistics indicate that alcohol consumption decreased in Finland by 9%, while little change was observed in Norway. In all surveys, reporting a decrease in alcohol use was more common than reporting an increase (ratios 2–2.6 in Finland, 1.3–2 in Norway). Compared to conventional surveys, in the ESAC survey fewer respondents reported no change and past‐year alcohol consumption was higher.

**Conclusion:**

The rapid survey using convenience sampling gave similar results on change in drinking as conventional surveys but higher past‐year drinking, suggesting self‐selection effects. Aspects of the pandemic driving alcohol consumption down were equally strong or stronger than those driving it up.

## 1 INTRODUCTION

The COVID‐19 pandemic hit the world in early 2020 and affected the lives and living conditions of most people in various ways, including availability and consumption of alcohol. The pandemic and its restriction can affect alcohol consumption in opposite ways, for example, poor economic situation reduces affordability; restrictions on bars, restaurants and entertainment venues reduce (heavy) drinking opportunities and on‐premise sales; and travel restrictions reduce travelers' alcohol imports; while increased stress, anxiety and loneliness may increase consumption in some segments of the population (Clay & Parker, 2020; Rehm et al., 2020).

For a basic understanding of how the pandemic has changed the population's alcohol consumption on average, reliable information on population‐level consumption change can in some jurisdictions be based on sales statistics, especially when complemented with other data sources on unrecorded alcohol consumption. Yet, even in countries where such reliable population‐level data are available, this data source cannot provide answers to questions about the degree of variation around the mean change, or whether different segments of the population, for example men and women or light and heavy drinkers, change in the same direction. To answer these questions, surveys are needed in addition to sales statistics.

In order to get a rapid assessment of changes in alcohol use in European countries, and as the available funding and time did not allow for a proper general population survey, data was collected from 21 European countries in the European Alcohol Use and COVID‐19 (ESAC) survey, a rapid online survey with various convenience sample data collection methods (Kilian, Manthey, Braddick, et al., 2020). The study found that in most of the countries, including all the Nordic countries, the proportion of respondents reporting a reduction in drinking quantity per occasion and in frequency of heavy episodic drinking events was larger than the proportion reporting an increase, and thus, a change score suggested an average reduction in overall alcohol consumption (Kilian et al., 2021). These findings are consistent with those of many other studies on changes in alcohol use during the COVID‐19 pandemic, where the majority found that the proportion of respondents reporting a decrease in their consumption was larger than the proportion reporting increased alcohol consumption (Alpers et al., 2021; Biddle, Edwards, Gray, & Sollis K, 2020; Bramness, Bye, Moan, & Rossow, 2021; Callinan et al., 2020; Chodkiewicz, Talarowska, Miniszewska, Nawrocka, & Bilinski, 2020; Manthey et al., 2020; Panagiotidis, Rantis, Holeva, Parlapani, & Diakogiannis, 2020; Sallie, Ritou, Bowden‐Jones, & Voon, 2020; Tran, Hammarberg, Kirkman, Nguyen, & Fisher, 2020), though some studies found more respondents increasing than decreasing their alcohol consumption (Georgiadou et al., 2020; Pollard, Tucker, & Green, 2020; Vanderbruggen et al., 2020). External validation of these findings by other data sources is rare, and it remains unclear whether—or to what extent—such survey reports are able to reflect an overall change in alcohol consumption (Kilian, Manthey, Probst, et al., 2020; Rehm, Kilian, Rovira, Shield, & Manthey, 2021).

Irrespective of the COVID‐19 pandemic, there is an increasing pressure to obtain data on health behaviours using faster and less expensive methods than those used to obtain traditional general population surveys employing probabilistic sampling. In addition to the costs, the traditional population surveys suffer to an increasing extent from non‐response, which may or may not be accompanied by increases in non‐response bias (Rehm et al., 2021). Alternative data collection methods include less expensive and faster surveys based on respondent panels, convenience samples and routinely collected data in registers and customer loyalty card databases. However, little is known about the extent to which findings from studies employing such alternative methods are biased due to issues such as self‐selected samples or data collection methods. It has therefore been suggested that findings from such surveys are triangulated and validated with external data (Rehm et al., 2021). In order to gain insight about the situations in which the savings in terms of time, cost, and effort is worthwhile, we need information from such validation studies (Mäkelä, 2021). In the present study, we validate findings from the ESAC survey for Finland and Norway, where other data sources are available for such validation.

Finland and Norway are two well‐off Nordic welfare states, Norway even more affluent than Finland, and they both have restrictive alcohol policies (Karlsson, 2014) and a fairly similar drinking pattern and level of alcohol consumption (Sierosławski et al., 2016). In the initial phase of the COVID‐19 pandemic, both countries employed relatively strict measures, though less strict than the curfews and stay‐at‐home‐orders seen in some other countries, and both experienced low infection rates in European comparison as well as low levels of morbidity and mortality due to COVID‐19 (Table 1). Norway offered more generous state compensation to industries and employees than Finland did. Moreover, while restaurants and bars were closed from the beginning of April until the end of May in Finland, selling alcohol was possible for premises serving food in Norway between March and June. These differences in measures implemented to curb the spread of the virus may have caused differences in both affordability and availability of alcohol in these two countries. Thus, although Finland and Norway are similar in several respects, factors driving alcohol consumption down (De Goeij et al., 2015; Rehm et al., 2020) would be expected to be somewhat stronger in Finland than in Norway.

**TABLE 1:** Restrictions applied due to the COVID‐19 pandemic in Finland and Norway in the spring and summer 2020

	Finland	Norway
Legal framework	The Emergency Powers Act.	The Infection Control Act.
Period of most restrictive policies	From mid‐Mar until mid‐Jun.	From 12 Mar 2020.
Restrictions:		
School and work	All schools from elementary schools (except the 1st and 2nd grades and classes for students with special needs) to universities moved to distance teaching for eight weeks beginning from mid‐Mar and working from home office was strongly recommended.	Lock‐downs of primary schools and kindergartens from mid‐Mar until the end of Apr and lock‐downs of junior high schools, high schools, University Colleges and Universities from mid‐Mar until mid‐May extensive use of home office for whom it was possible.
Restaurants and bars	Closed from the beginning of Apr until the end of May	Between 12 Mar and 1 Jun: Alcohol sale restricted to premises with serving of food (not buffet) and where visitors and personnel could keep at least 1 m distance.
Social gatherings	Social gatherings with more than 10 people were prohibited. Many public premises like theatres, museums, libraries, and sports facilities closed.	Restrictions on social gatherings such as cultural and sports events, organized sports.
Travel	Passenger traffic to Finland was suspended until mid‐May. Restrictions on traffic out of the Helsinki‐Uusimaa region.	Strict travel restrictions between mid‐Mar and mid‐Jun.
Other		The degree of restrictions varied, with the most restrictive measures implemented in the capital of Norway (Oslo).

Against this back‐drop, we aim to examine two intertwined issues using comparable data in these two countries. Our methodological aim is to assess the validity of the rapid web‐based ESAC survey's results and thereby to contribute to the accumulation of knowledge about the validity and reliability of rapid data collection methods. We pursue this aim by comparing the findings from the ESAC survey with findings from two different types of data sources in Finland and Norway. On the one hand, we use data from general population surveys conducted at about the same time using more conventional sampling techniques and comparable measures on the level of alcohol use (for Norway) and change in alcohol use (Norway and Finland) during the first months of the COVID‐19 pandemic. In the surveys, the measures about change in consumption are based on the respondents' retrospective self‐report about change in alcohol use, and the aggregate of such self‐reports do not necessarily reflect the overall change in consumption, for instance due to sampling bias, inaccurate measures of change, and reporting bias (Blome & Augustin, 2015). Therefore, we also examine change in aggregate‐level measures of total per capita alcohol consumption and its change, based on recorded sales data and estimates of unrecorded consumption.

Our substantive aim is to assess changes in alcohol consumption in Finland and Norway during the first months of the COVID‐19 pandemic using the best available data sources. We consider per capita alcohol consumption estimates to be the most reliable source about aggregate‐level change, but information about variation around the mean change, that is, how common it was that consumption increased or decreased, can only be obtained from surveys (Mäkelä, 2021; Rehm et al., 2021). In this study, we use both data sources to examine changes in alcohol consumption in Finland and Norway during the first months of the COVID‐19 pandemic.

To summarize, the aims were:

(1) Methodological: to examine how a rapid data collection fares in estimating change in alcohol consumption when compared to more conventional sources of information.
(2) Substantive: to examine how alcohol consumption changed in Finland and Norway during the first months of the COVID‐19 pandemic.

## 2 METHODS

### 2.1 The ESAC web‐based survey

Finland and Norway were included in the ESAC survey, which was conducted to gather information on changes in alcohol consumption among adults aged 18 years or older during the COVID‐19 pandemic in Europe (www.covid19‐and‐alcohol.eu). The survey was conceptualized in English, translated into 20 languages including Norwegian and Finnish, and disseminated in 21 European countries. Different sampling techniques were employed, aiming to collect data from a convenience sample from the society as widely as possible. Respondents were in both countries recruited from alcohol research and policy networks, social media and web pages of the collecting organizations. In Norway, the most successful recruitment channel was one online version of a large national newspaper, and in Finland a paid Facebook advertisement. Details concerning study design and implementation have been published in a separate study protocol (Kilian, Manthey, Braddick, et al., 2020). The study was in the field from the end of April to the end of June 2020 in both countries. Since the sample population did not match the actual population of the country due to convenience sampling, sampling weights were calculated. The sampling weights adjusted the skewed sampling distribution to the actual country population (Eurostat, 2020) according to gender, age group and educational attainment (for details, see Kilian, 2020). All surveys were weighted to restore population representation and thus ensure better comparability. Basic information on all surveys, including the variables calculated in the applied weights, is shown in Table 2.

**TABLE 2:** Sampling, sample characteristics and measurement of change in alcohol consumption under the pandemic in population surveys in Finland and Norway

	ESAC	Finnish population survey	Norwegian web Panel survey	Norwegian population survey	Norwegian regional survey
Target population	Adults 18+, national	Adults 18‐69, national	Adults 18+, national	Adults 16–79, national	Adults 18+, regional area^a^
Sampling^b^	Convenience	Probability	Convenience	Probability	Probability
Collection	Web survey	Web survey	Web panel	CATI	Web survey
Response rate (if applicable)	NA	44%	27%	60%	36%
Net sample	Finland: 3800 Norway: 17,092	2345	1328	2189	25,708
Weighted by^c^	Gender, age, education	Gender, age, region	Gender, age, region	Gender, age, education, region	Gender, age, education
Past‐year alcohol consumption	AUDIT‐C	NA	AUDIT‐C	AUDIT‐C	AUDIT‐C
Change in drinking	Graded measures^d^	Crude measures^e^	Graded measures^f^	NA	Crude measures^g^

Abbreviation: CATI, Computer Assisted Telephone Interview.

^a^ Bergen municipality (second largest city in Norway).

^b^ For Norwegian probability samples, the samples were simple random samples. For the Finnish probability sample, random sampling of individuals within five selected representative regions was carried out.

^c^ For more information about weighting procedures, see Alpers et al., 2021; Bramness et al., 2021; Kilian, Manthey, Braddick, et al., 2020; Mäkelä et al., 2020; Torsteinsen, 2020.

^d^ In the past month, did] (i) you drink alcohol less or more often? (ii) the amount of alcohol you usually drink on each drinking occasion (i.e., the volume of alcohol consumed) change? (iii) the frequency of drinking occasions where you drank a high amount of alcohol (i.e., six or more drinks) change? Response options: (1) Drink much less often/Drink much less; (2) Drink slightly less often/Drink slightly less; (3) No change; (4) Drink slightly more often/Drink slightly more; (5) Drink much more often/Drink much more.

^e^ Has the corona pandemic or the measures applied to limit it had an effect on… your alcohol use? Response options: (1) no effect, (2), yes, reduced, (3) yes, increased, (4) doesn't apply.

^f^ During the pandemic] have you drunk more, less, or about the same as you did before the pandemic? Response options: ‘Much less’, ‘A little less’, ‘About the same’, ‘A little more’ and ‘Much more’.

^g^ How has your alcohol consumption changed during the period of pandemic measures? Response options: ‘decreased’, ‘not changed’ or ‘increased.

### 2.2 The Finnish general population survey

A general population survey was conducted as a part of a serological population study of the COVID‐19 pandemic. The study was conducted by the Finnish Institute for Health and Welfare, and it was based on random sampling within five hospital districts in the population aged 18–69. Each of the five districts had been chosen to represent one of the five catchment areas in different parts of Finland. The study is on‐going, with new respondents every week. Here we apply data from the 2345 respondents who participated in the period May 4th–June 30th. The response rate was 44%. The question on change in alcohol use (presented in the questionnaire within a list of 19 different health behaviours and living conditions) is shown in Table 2. The response options 1 (no effect on alcohol use) and 4 (does not apply to me) were combined in reporting.

### 2.3 The Norwegian surveys

Data from three surveys, all conducted in the second quarter of 2020, were available. As all three surveys comprised identical measures on past‐year alcohol consumption to that of the ESAC survey, but varied with regard to sampling and data collection method, each survey contributed to the validation of the ESAC survey (see Table 2). Two of these surveys also included measures of change in alcohol consumption during the pandemic.

Web panel survey: conducted by the opinion poll ‘Opinion’ on behalf of the Norwegian Directorate of Health in June 2020. The survey was sent to 4844 Norwegians aged 18 years and older, randomly selected from a national web panel (a convenience sample) and was completed by 1328 respondents (27.4%). The question asked is shown in Table 2.

Population survey: conducted by Statistics Norway in April–June 2020, applying a simple random sample of the population aged 16–79 years, and data were collected by computer‐assisted telephone interviews (Torsteinsen, 2020). The response rate was 60%, and the net sample comprised 2189 respondents (see Table 2 for questions posed).

Regional survey: conducted in the second half of April 2020. A simple random sample of 81,000 adult (aged 18 and older) residents in the city of Bergen was extracted from the population registry and contacted via SMS and e‐mail. Data collection was web‐based and the survey was completed by 25,708 respondents (36%) (see Table 2 for questions posed). Available data from this survey is limited to those published (Alpers et al., 2021).

### 2.4 Survey measures and statistical analysis

In all surveys, the estimate of change in alcohol use was based on a retrospective self‐report of the change. In the ESAC survey, questions were posed separately on changes in drinking frequency, typical quantity per occasion, and heavy episodic drinking, while in the other surveys an overall question about change in drinking was posed (Table 2).

Comparison between surveys was done using three different indices for the ESAC survey. Index 1 applied a sum score for the three change items. For each, a value of 2 was given to ‘much change’, 1 for ‘slight change’ and 0 for ‘no change’—negative for decreases and positive for increases. Positive scores were categorized as an increase, negative scores as a decrease, and score 0 was marked as no change.

Index 2 was based solely on questions on changes in drinking frequency and quantity per occasion. ‘No change’ included those with ‘no change’ on both, ‘no change’ on one and a ‘slight change’ on the other, or ‘slight change’ in opposite directions. ‘Decrease’ included those with ‘much less’ on one and ‘much less’, ‘slightly less’ or ‘no change’ on the other, or ‘slightly less’ on both. ‘Increase’ included those with ‘much more’ on one and ‘much more’, ‘slightly more’ or ‘no change’ on the other, or ‘slightly more’ on both.

Index 3 was based only on responses to changes in drinking frequency and collapsed into three categories.

*Past‐year alcohol consumption* was assessed with the three AUDIT‐C items (frequency of drinking, usual quantity per occasion, and frequency of drinking 6+ units per occasion) which referred to a time window of 12 months preceding the survey (for details, see Kilian, Manthey, Braddick, et al., 2020). The frequency of drinking and the usual quantity per occasion variables were transformed into semi‐continuous measures taking the mid‐point values of the categories, and from these, we calculated alcohol consumption in number of units consumed per week.

Differences in means for drinking frequency, quantity per occasion, and units per week between the ESAC survey and the other surveys were tested by two‐sample *t*‐tests. All surveys were weighted to restore population representation and thus ensure better comparability (see Table 2).

### 2.5 Data on total per capita alcohol consumption

*Finland*. We used data compiled by the Finnish Institute for Health and Welfare (Mäkelä et al., 2020) to estimate the change in total per capita alcohol consumption, combined from two main sources for the second quarter in 2019 and 2020: (1) recorded sales data for all off‐premise and on‐premise sales, and (2) estimates of unrecorded alcohol consumption based on travelers' alcohol imports and online purchases from abroad, both measured in litres of pure alcohol per inhabitant 15 years and over. Data for the second category was obtained from weekly general population surveys of self‐reported alcohol imports for the previous two weeks (*n* = 26,000 per year) (THL, the Finnish Institute for Health and Welfare, 2021). Due to too much random variation in the monthly series, the annual change in alcohol imports in 2020 was divided to months using changes in the number of travelers to Finland, based on national statistics. These data on the amount of travelers' alcohol imports is a good match with estimates of tourists' purchases made in Estonia, the main source of travelers' imports to Finland (Eesti Konjunktuuriinstituut, 2020, S. 30). Hence, we consider the rolling survey, with only a 14‐days reference period for travelers' imports, to tackle problems of underestimation sufficiently.

*Norway*. Also for Norway, estimates of the change in total per capita consumption were based on two main sources for the second quarter in 2019 and 2020: (1) recorded sales data for all off‐premise and on‐premise sales (Statistics Norway, 2021b), and (2) estimates of unrecorded consumption, both measured in litres of pure alcohol per inhabitant 15 years and over. Estimates of unrecorded consumption are based on two data sources: (i) registered sales data for tax‐free alcohol purchases at arrivals from abroad in Norwegian airports (accounting for about half of all unrecorded consumption), and (ii) general population survey data on cross‐border trade and other tourist imports in the 12 months preceding the survey (NIPH, the Norwegian Institute of Public Health, 2021). We assumed that unrecorded consumption accounts for approximately the same share of total consumption in the 2nd quarter of 2019 as in a full year. Monthly data on aviation traffic from abroad (Avinor, 2021) and quarterly data on cross‐border shopping (Statistics Norway, 2021a) show that these activities were reduced by around 98%–99% between the comparison periods, and thus suggest that unrecorded consumption in the 2nd quarter of 2020 was close to zero. It should be noted that survey estimates of unrecorded consumption (category ii above) collected in this way are likely downward biased in the same manner as consumption estimates, which often capture clearly less than half of total per capita alcohol consumption (Kilian, Manthey, Probst, et al., 2020). We made a sensitivity analysis for the Norwegian data in which the change in consumption was estimated also using the assumption that unrecorded consumption estimated from the survey covered half of the real amount.

## 3 RESULTS

### 3.1 Did ESAC capture overall changes in alcohol consumption?

Table 3 shows the changes in recorded alcohol sales, estimated unrecorded consumption and estimated total alcohol consumption between the 2nd quarters in 2019 and 2020.

**TABLE 3:** Observed changes in total per capita (15 years and older) consumption by country

	Finland			Norway		
	2019	2020	Change	2019	2020	Change
Recorded sales 2nd quarter, litres per capita	2.21	2.26	+2%	1.61	1.94	+20%
Estimated unrecorded consumption in litres (% of total)^a^	0.48 (18%)	0.19 (8%)	−60%	0.20 (11%)	0.0 (0%)	∼−100%
Estimated total consumption 2nd quarter, litres per capita^a^	2.69	2.45	−9%	1.81	1.93	+7%
Estimated unrecorded consumption in litres (% of total)^b^				0.30 (16%)	0.0 (0%)	∼−100%
Estimated total consumption 2nd quarter, litres per capita^b^				1.91	1.93	+1%

Abbreviation: ESAC, European Alcohol Use and COVID‐19.

^a^ Estimates of unrecorded consumption and total consumption assuming complete coverage of unrecorded consumption in surveys.

^b^ Estimates of unrecorded consumption and total consumption assuming 50% coverage of unrecorded consumption in surveys. In Finland, unrecorded consumption is estimated from rolling surveys covering 2‐week periods; in Norway, half of estimated unrecorded consumption is based on a survey covering a 12‐month period.

For Finland, official statistics indicate that the recorded alcohol sales increased by 2%. However, travellers' alcohol imports and Internet purchases from abroad, which made almost a fifth of total consumption in 2019, were estimated to have decreased by approximately 60%, from 0.48 to 0.19 litres per capita. When combined, total per capita consumption was estimated to have decreased by 9% in the second quarter of 2020 compared to the same time period in 2019.

For Norway, recorded sales in the 2^nd^ quarter increased by 20% from 2019 to 2020. Estimated unrecorded consumption corresponded to 11% of the estimated total consumption in 2019, and it was estimated to have almost completely ceased, decreasing from 0.2 to 0 L of alcohol per capita. This implies an increase in total alcohol consumption by almost 7%. However, according to the sensitivity analysis which assumes that the survey estimate covers only half of the real amount of unrecorded consumption, total alcohol consumption was likely to be fairly similar in the second quarters of 2019 and 2020.

Table 4 shows the ESAC survey results for Finland and Norway. Depending on how change in consumption was operationalized for analysis, in Finland there were 2.0–2.6 times more respondents who reported a decrease compared to those who reported an increase in alcohol consumption. In the Norwegian ESAC data, there were also more respondents reporting a decrease than an increase, though the ratio was somewhat lower, that is, 1.3–2.0. The ratios were smallest for the third, least fine‐grained indicator covering only frequency of drinking, and larger for the indicators including also typical quantity (indicator 2) and typical quantity and HED (indicator 3), which was also the most fine‐grained indicator.

**TABLE 4:** The proportion of respondents reporting changes in alcohol consumption in the early phase of the COVID‐19 pandemic by survey and country, among past‐year drinkers, by country

	Finland				Norway				
	ESAC (*n* = 3456)			National survey	ESAC (*n* = 15,349)			Web panel survey	Regional survey
	Ind1	Ind2	Ind3	(*n* = 2345)	Ind1	Ind2	Ind3	(*n* = 1195)	(*n* = 23,319)
Reported change (%)									
No change	38.5	57.0	44.4	78.2	41.0	63.8	48.8	57.4	67.4
Decrease	44.4	30.7	37.1	14.0	39.4	24.0	28.9	29.9	20.5
Increase	17.1	12.2	18.5	7.8	19.6	12.2	22.4	12.8	12.1
Ratio									
Decrease:increase	2.6	2.5	2.0	1.8	2.0	2.0	1.3	2.3	1.7

*Note*: The categorization of responses to change in drinking behaviour in ESAC was conducted using three approaches. Index 1 (Ind1) applied a sum‐score for changes. Index 2 (Ind2) was based solely on responses to changes in drinking frequency and quantity per occasion with only slight changes combined to the ’no change’ category. Index 3 (Ind3) was based only on responses to changes in drinking frequency and collapsed into three categories.

### 3.2 Comparison of the change in alcohol consumption in the different surveys

Table 4 shows that the distribution of the change categories (no change/decrease/increase) differed not only between the three indicators used in the ESAC survey but also across the surveys. The prevalence of the ‘no change’ category was, as could be expected for purely technical reasons, lowest for the most fine‐grained indicator 1, but even when the estimated change was based on frequency of drinking with its three response categories only (indicator 3), the proportion of the ‘no change’ category was lower for the ESAC survey than for the surveys using more conventional methods.

In all surveys, the proportion reporting a decrease in alcohol use was substantially larger compared to the proportion reporting an increase. Here, the ESAC survey did not systematically differ from others: in Finland, the ratio between the proportion decreasing and the proportion increasing their consumption was higher in the ESAC survey than in the national survey; in Norway, the ratio in the ESAC survey was, for two indicators out of the three, in between those in the two other surveys.

### 3.3 Comparison of past‐year alcohol consumption in the different surveys

The Norwegian data can be used to compare the results from different surveys regarding the level and pattern of reported past‐year alcohol consumption, based on the three AUDIT‐C items (Table 5). There were marked differences between ESAC and the other surveys. The surveys furthest apart from each other were ESAC on the one hand and the population survey based on probability sampling and telephone interviews on the other. In the probability survey, as compared to the ESAC survey, the proportion of abstainers was higher, and among drinkers the drinking frequency, the usual quantity per occasion, and thus also the estimated weekly consumption was substantially lower. While the proportion of abstainers did not differ substantially between the ESAC and the Norwegian web panel survey or the Norwegian regional survey, the estimated weekly consumption was higher in the ESAC survey.

**TABLE 5:** Past‐year alcohol consumption by data source, Norway

	ESAC (*n* = 17,137)	Web panel survey (*n* = 1328)	Population survey (*n* = 2119)	Regional survey (*n* = 25,708)
Proportion of abstainers, %	9.3	10.0	14.9	9.0
Past‐year alcohol consumption (past‐year drinkers only)	*n* = 15,549	*n* = 1195	*n* = 1803	*n* = 23,319
Frequency/year (SD)	64.3 (68.6)	63.5* (65.5)	46.6** (54.7)	NA
Quantity/occasion, units (SD)	4.2 (3.1)	3.0 ** (2.2)	3.1 ** (2.3)	NA
Volume: Weekly units (SD)	5.5 (8.6)	3.7 ** (5.1)	2.8 ** (4.6)	3.2 ** (4.8)
HED frequency/year^a^, %	34	31	18	14

^a^ Drinking more than six units on one occasion at least once a month.

*p*‐values for differences: *p = 0.018, **p < 0.001.

## 4 DISCUSSION

Our results based on national sales statistics, combined with data on change in unrecorded consumption, showed that in Finland alcohol consumption has clearly decreased, while in Norway there has most likely been little change in per capita alcohol consumption during the initial stage of the COVID‐19 pandemic. In all surveys, the proportion of respondents reporting decreased alcohol consumption was clearly larger than the proportion reporting increased consumption, and the ratio was greater in Finland than in Norway. The ratio was not systematically higher or lower in the ESAC survey compared to the other surveys, but in the conventional surveys using probability sampling, there were more respondents reporting no change in alcohol use and the level of the volume of alcohol consumed was lower.

We believe that the figures based on sales statistics and unrecorded consumption give the best estimate of the changes in per capita alcohol consumption, even though estimates of unrecorded consumption can never be fully accurate. There is greater reason to believe the unrecorded consumption (and therefore also its change) to be underestimated in Norway than in Finland. This is because one‐half of the Norwegian estimate is derived from an annual survey asking respondents to estimate unrecorded consumption over 12 months, while the Finnish data comes from a rolling survey asking about a period of two weeks, and clearly higher estimates on imports to Finland would leave implausibly low amounts of alcohol to be used by Estonians. Therefore, we would take the Finnish figures at their face value (a 9% decrease in total consumption) but would consider the sensitivity analysis to be a more likely depiction of the real development in Norway. That is, a very small change in per capita alcohol consumption is likely to have occurred.

The figures based on sales statistics and unrecorded consumption should roughly equal aggregated changes of individuals' *volume* of consumption. In principle, stockpiling and changes in storage of alcohol could cause these to differ. We have no evidence of changes in storage of alcohol for Finland or Norway, but UK evidence has been interpreted to signal that stockpiling was unlikely (Anderson, Llopis, O'Donnell, & Kaner, 2020). However, an important aspect causing a difference between survey results and the statistics is that all the surveys measured the *proportion* of respondents retrospectively reporting changes in drinking and not changes in volume of consumption. For Norway, the two sets of results seem contradictory (not much change vs. clearly more people reporting a decrease than an increase), but both can be true if lighter drinkers decreased and heavier drinkers increased their consumption. Recent findings from Norway support this notion, that is, for most respondents, an average modest decline in consumption was found. However, the small fraction with the highest pre‐pandemic consumption increased their consumption substantially, and in effect, the proportion of heavy drinkers increased markedly (Rossow, Bye, Moan, Kilian, & Bramness, 2021; see also Manthey et al., 2020). Thus, it is essential to take into account—also when interpreting results from other surveys—that a slight decrease of a heavy drinker may far exceed a major decrease of a light drinker. Ideally, this should also be reflected in what is measured in surveys.

Other methodological explanations for the difference between the estimates based on the statistics and the surveys cannot be ruled out. The first important limitation of surveys is the reliance on self‐reports, as alcohol consumption is usually underreported in surveys when compared with more reliable data sources (Kilian, Manthey, Probst, et al., 2020). It is likely that underreporting may also have an impact on self‐reported consumption change. Some of this underreporting may be due to social desirability bias among the respondents (Davis, Thake, & Vilhena, 2010; Krumpal, 2013), or a low coverage of heavy drinking populations (Shield & Rehm, 2012). Self‐selected surveys (convenience samples) can be of advantage here, as they may attract the attention of the relevant consumer group, particularly if the survey is about a sensitive topic such as alcohol consumption (Krumpal, 2013). A second important limitation in the survey measurement in both our surveys and most of those used in the literature so far is that it was based on *retrospective* assessment of change. We do not know the distribution of self‐reported change in a situation without the pandemic and what part of the change is specific to the pandemic situation. Additionally, retrospective self‐assessments of changes have been reported to have their own biases beyond the social desirability effect (Blome & Augustin, 2015).

Variation around the mean change in alcohol consumption, that is that everyone did not act the same way, can only be measured and understood using surveys. Consistent with findings in most other studies (Alpers et al., 2021; Biddle et al., 2020; Bramness et al., 2021; Callinan et al., 2020; Chodkiewicz et al., 2020; Manthey et al., 2020; Panagiotidis et al., 2020; Sallie et al., 2020; Tran et al., 2020), this study and the several surveys we used showed that also in Finland and Norway the proportion reporting a decrease in alcohol use during the first months of the COVID‐19 pandemic was larger than the proportion reporting an increase in alcohol use. In the society, there are always simultaneously factors that tend to drive individuals' and the society's consumption upwards and those that drive it downwards (Room, Österberg, Ramstedt, & Rehm, 2009). In the pandemic, the former may have included increased stress, anxiety and loneliness, and the latter reduced availability in on‐premise salespoints and from abroad, or reduced affordability and social functions (Clay & Parker, 2020; Rehm et al., 2020). Aching to this, de Goeij et al. (2015) have concluded that economic crises affect alcohol consumption and related harm through two opposing mechanisms: reductions in alcohol consumption due to tighter budget constraints and a rise in harmful drinking due to increased psychological distress. Clearly, in Finland and Norway the factors driving consumption down during the pandemic outweighed the factors driving consumption up for more people than for whom the contrary was true, and in Finland this applied even when the total litres of changes are considered. A similar decrease in per capita consumption (and related harm) occurred in Finland in the early 1990s severe depression (Valkonen, 2000). The difference between Finland and Norway could partly be due to more generous state compensations to industries and employees in Norway than in Finland. In addition, while restaurants and bars in Finland were closed in the early phase of the pandemic, sale of alcohol was allowed for premises serving food in Norway in the same period. Hence, both affordability and availability of alcohol was greater in Norway than in Finland, thus offering some potential explanations of the country differences observed in change of alcohol consumption in these two countries. However, a more detailed analysis of the intervening factors and their effects on different countries and population groups during the pandemic remains a task for future research.

Further insight on our methodological aim, to examine how a rapid data collection (ESAC) fares in comparison to other data sources, was obtained by comparing results between different types of surveys. Regarding the estimation of change in alcohol use, the overall picture was similar in the ESAC survey collected using convenience samples and in the surveys using probability samples: far more people reported decreased than increased alcohol use. The ratio between these groups was not systematically higher or lower in the rapid survey compared to the other surveys.

The fact that past‐year alcohol consumption was the highest in the ESAC survey suggests that the convenience sample may disproportionately have attracted people who drink more, possibly reflecting more interest in alcohol‐related topics. Our result showing that the proportion of respondents reporting no change in their alcohol use during the pandemic was low in the ESAC survey suggests a similar self‐selection: people whose consumption was affected by the pandemic may have disproportionately volunteered to the convenience sample. When comparing past‐year alcohol consumption, the web‐panel survey, which was also based on a convenience sample, was in between the ESAC survey and the surveys based on probability samples. This suggests that similar self‐selection mechanisms that apply to the ESAC survey's ad hoc convenience sample may also apply to the more permanent response panels and/or the selection within them to respond to this particular survey. As indicated above, such a self‐selection does not automatically imply a bias for the worse. For sensitive topics such as alcohol consumption, web‐based self‐selected surveys may help in reducing the problem of under‐estimation of alcohol use. However, systematic research on this is missing so far. Self‐selection could affect the constitution of the samples also in other ways, for example, active Internet users or people with more spare time can participate disproportionately in self‐selected samples, and if there is a systematic difference in alcohol consumption along these aspects, this would also have an impact on measures of alcohol use or its change.

The general population surveys based on probability samples are not free of biases, either. They miss large parts of the original samples, so the difference between the types of surveys could be partly due to heavier drinkers disproportionately dropping out from the general population surveys. Additionally, it is known that survey modes relying on respondent self‐administration (like web surveys) obtain greater reports of substance use than modes in which the interviewer asks about substance use (Johnson, 2014). Finally, although all survey data were weighted, we cannot rule out possible biases in the consumption distribution (Kilian, Manthey, Probst, et al., 2020) and reported changes in consumption during the pandemic in Norway and Finland.

Obviously, there is no winner among surveys in assessing alcohol use in the general population, since probabilistic as well as non‐probabilistic surveys are subject to bias. Which approach should be taken depends on the aim of the study, available resources, target sample, and what the survey is about. For example, if the aim is to assess associations between recent changes in drinking behaviour and responses to a sudden unexpected event such as the COVID‐19 pandemic, a rapid web survey may be preferable. On the other hand, if the aim is to assess whether or to what extent the alcohol consumption level changes over time in various demographic strata, surveys using probability samples are the most suitable. There is an extensive body of research that attempts to identify which methodology is best suited to capture self‐reported alcohol consumption under different scenarios, constituting an important contribution to our field (e.g. Ekholm, Strandberg‐Larsen, & Grønbæk, 2011; Greenfield & Kerr, 2008; Heeb & Gmel, 2005; Nugawela, Langley, Szatkowski, & Lewis, 2016). However, survey methodology seems to explain only little variance in underreporting of alcohol consumption across surveys and countries (Kilian, Manthey, Probst, et al., 2020). As is done in this study, further comparison of different surveys and data sources is needed to get a more comprehensive picture of how and under which circumstances alcohol consumption changes (see also Mäkelä, 2021; Rehm et al., 2021).

Limitations of the current study include that it is a comparative secondary data analysis rather than a specifically designed, rigorous methodological study, and therefore the questions were not identical in the different surveys. Moreover, sampling methods and data collection methods varied across the available data sets; they were included for this reason, as triangulation was our aim, but differences in many aspects makes it difficult to pinpoint the exact cause for differences. Finally, the uncertainty about unrecorded consumption in assessing total per capita alcohol consumption needs to be acknowledged.

In conclusion, in Finland the pandemic reduced the volumes of alcohol consumed, while in Norway the increased recorded alcohol sales seemed to offset the decreases in travelers' imports. Methodologically, it seems that the ESAC survey, based on convenience samples, managed to measure retrospectively self‐reported change in alcohol use at a par with studies using probability‐based sampling. It should be noted, though, that regardless of the type of survey data collection used, it is important to carefully consider the indicator measured (e.g. proportion increasing or decreasing vs. quantification of the change in volume of consumption) and the possibility that all surveys relying on retrospective self‐reports of change can be biased. The *level* of alcohol use varied across survey types, but interpretation of what causes this and which survey is more reliable than another is challenging.

## CONFLICT OF INTERESTS

Pia Mäkelä and Kirsimarja Raitasalo would like to declare that their institute has received funding for a research project, of which the current work is a part, and for some data collections from Alko Ltd, the Finnish state‐owned alcohol retail monopoly. The other authors have no conflict of interests to declare.

## Data Availability

The data that support the findings of this study derives from different sources and is therefore subject to varying degrees of accessibility. The ESAC survey is openly available in the Figshare repository at https://doi.org/10.6084/m9.figshare.13580693.v1. Access to the national data can be requested from the corresponding author.
